# Lipoprotein(a) Inhibition: Current Evidence, Therapeutic Landscape, and Future Directions

**DOI:** 10.31083/RCM55004

**Published:** 2026-08-28

**Authors:** Pierre Sabouret, Domenico Mario Giamundo, Joanna Popiolek-Kalisz, Luigi Spadafora, Marco Bernardi, Elad Asher, Mamas A Mamas, Dan Atar, Giuseppe Andò

**Affiliations:** ^1^ACTION Study Group, Inserm UMRS1166, Heart Institute, Pitié-Salpetriere Hospital, Sorbonne University, 75013 Paris, France; ^2^Division of Cardiology, Policlinico Casilino, 00169 Rome, Italy; ^3^Clinical Dietetics Unit, Department of Bioanalytics, Medical University of Lublin, 20-059 Lublin, Poland; ^4^Department of Cardiology, Cardinal Wyszynski Hospital in Lublin, 20-718 Lublin, Poland; ^5^Department of Clinical, Internal Medicine, Anesthesiology and Cardiovascular Sciences, Sapienza University of Rome, 00185 Rome, Italy; ^6^Jesselson Heart Center, Shaare Zedek Medical Center, The Eisenberg R&D Authority, and Faculty of Medicine, Hebrew University of Jerusalem, 9103102 Jerusalem, Israel; ^7^Keele Cardiac Research Group, Centre for Prognosis Research, Keele University, ST5 5BG Stoke-on-Trent, UK; ^8^Department of Cardiology, Oslo University Hospital, 0450 Oslo, Norway; ^9^Institute of Clinical Medicine, University of Oslo, 0372 Oslo, Norway; ^10^Department of Clinical and Experimental Medicine, University of Messina and Azienda Ospedaliera Universitaria Policlinico “Gaetano Martino”, 98125 Messina, Italy

**Keywords:** lipoprotein(a), atherosclerosis, cardiovascular diseases, aortic valve stenosis, antisense oligonucleotides, small interfering RNA

## Abstract

Lipoprotein(a) [Lp(a)] is a genetically determined, apolipoprotein B-containing lipoprotein that contributes to cardiovascular risk through atherogenic, proinflammatory, and potentially antifibrinolytic mechanisms. Genetic, epidemiological, and mechanistic evidence supports a causal role for elevated Lp(a) in atherosclerotic cardiovascular disease and calcific aortic valve disease. In contrast, associations with other cardiovascular phenotypes are less consistent. Since circulating concentrations are largely inherited and only modestly affected by lifestyle measures and conventional lipid-lowering therapies, Lp(a) represents an important component of residual cardiovascular risk. This review summarizes the structural, genetic, and pathogenic characteristics of Lp(a), examines the strength of evidence across coronary artery disease, ischemic stroke, peripheral artery disease, abdominal aortic aneurysm, calcific aortic valve disease, and heart failure, and discusses measurement, clinical interpretation, and potential biomarkers. The therapeutic landscape has expanded rapidly. Antisense oligonucleotides and small interfering RNA agents substantially suppress hepatic apolipoprotein(a) synthesis, oral small-molecule inhibitors prevent Lp(a) particle assembly, and *in vivo* gene-editing strategies aim to provide highly durable reductions. Early-phase trials have demonstrated marked lowering of circulating Lp(a); however, most were not designed to assess cardiovascular outcomes, so direct comparisons among agents are limited by differences in study populations, assays, dosing schedules, and follow-up. Ongoing cardiovascular outcome trials will determine whether selective Lp(a) lowering reduces clinical events and which patients are most likely to benefit. Until these results are available, Lp(a) measurement can refine risk stratification and support intensive management of modifiable risk factors, whereas biomarker reduction alone should not be equated with proven clinical benefit.

## 1. Introduction

Lipoprotein(a) [Lp(a)] is a genetically determined, apolipoprotein B (apoB)-containing lipoprotein composed of a low-density lipoprotein-like particle covalently bound to apolipoprotein(a) [apo(a)]. Through its atherogenic, proinflammatory, and antifibrinolytic properties, Lp(a) contributes to cardiovascular risk independently of, and in addition to, low-density lipoprotein cholesterol (LDL-C). Genetic, epidemiological, and mechanistic evidence supports a causal role for elevated Lp(a) in atherosclerotic cardiovascular disease and calcific aortic valve disease [1,2,3].

Despite this evidence, Lp(a) measurement has historically received limited attention in routine cardiovascular risk assessment, partly because circulating concentrations are largely genetically determined, lifestyle interventions have little effect, and no specific pharmacological treatment was available. The development of therapies directly targeting apolipoprotein(a) synthesis has now renewed clinical and scientific interest in Lp(a), with several agents achieving marked and sustained reductions in circulating concentrations.

This review summarizes the biological and pathogenic characteristics of Lp(a), examines its clinical relevance across different cardiovascular phenotypes, and critically discusses the efficacy, limitations, and potential clinical positioning of emerging Lp(a)-lowering therapies. Particular attention is given to ongoing cardiovascular outcome trials, which will determine whether profound Lp(a) reduction translates into improved clinical outcomes and can be incorporated into future cardiovascular prevention strategies (Fig. 1).

**Fig. 1.**
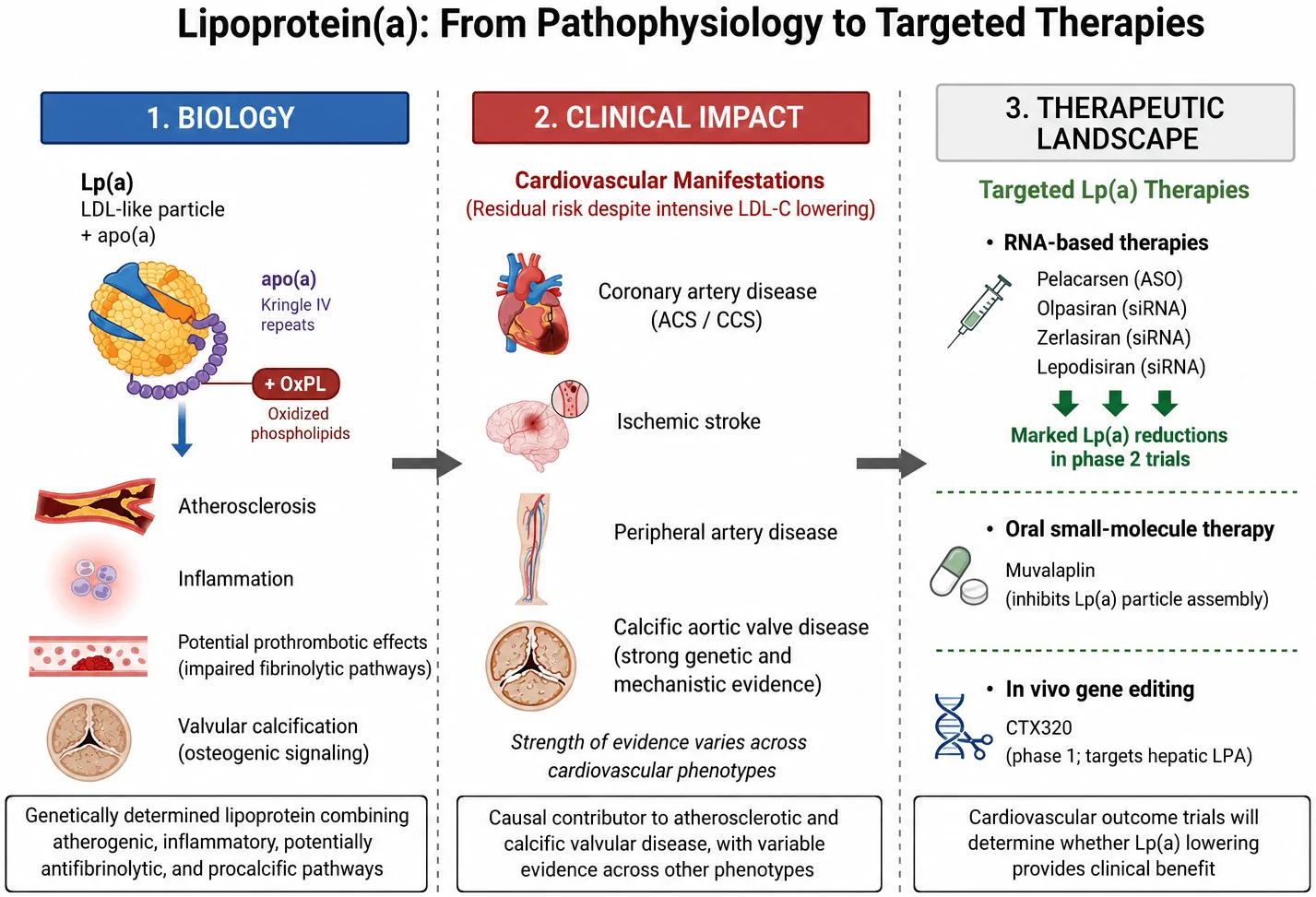
**Lipoprotein(a): from pathophysiology to targeted therapies**. Lipoprotein(a) [Lp(a)] is a genetically determined lipoprotein composed of an apolipoprotein B-containing low-density lipoprotein-like particle covalently linked to apolipoprotein(a). Its pathogenic effects arise from the combination of arterial deposition of an atherogenic particle, carriage of oxidized phospholipids, vascular inflammation, potentially impaired fibrinolytic pathways, and promotion of osteogenic signaling within the aortic valve. Elevated Lp(a) is causally implicated in atherosclerotic cardiovascular disease and calcific aortic valve disease, although the strength of evidence varies across other cardiovascular phenotypes. Investigational treatments include antisense oligonucleotides and small interfering RNA agents that suppress hepatic apolipoprotein(a) synthesis, the oral small-molecule muvalaplin that inhibits Lp(a) particle assembly, and the *in vivo* gene-editing therapy CTX320 targeting hepatic LPA. Although these approaches achieve marked reductions in circulating Lp(a), ongoing cardiovascular outcome trials are required to determine whether biomarker lowering translates into clinical benefit. ACS, acute coronary syndrome; apo(a), apolipoprotein(a); apoB, apolipoprotein B; ASO, antisense oligonucleotide; CCS, chronic coronary syndrome; LDL-C, low-density lipoprotein cholesterol; *LPA*, apolipoprotein(a) gene; Lp(a), lipoprotein(a); OxPL, oxidized phospholipids; siRNA, small interfering RNA. The figure is an original work created by the authors and has not been reproduced or adapted from previously published material.

## 2. Biological and Pathophysiological Features of Lp(a)

### 2.1 Structure and Function

Lipoprotein(a) consists of a low-density lipoprotein-like particle containing apolipoprotein B-100 (apoB-100), covalently linked by a disulfide bond to apo(a). Apo(a) contains multiple kringle IV (KIV) domains, including a highly variable number of kringle IV type 2 (KIV-2) repeats. This copy-number variation determines apo(a) isoform size and is inversely associated with circulating Lp(a) concentrations. Because apo(a) shares structural homology with plasminogen, Lp(a) may interfere with fibrinolytic pathways and contribute to a prothrombotic milieu [1,2].

In addition to its cholesterol-rich apolipoprotein B-containing core, Lp(a) is a major carrier of oxidized phospholipids (OxPL). These bioactive lipids promote endothelial activation, monocyte recruitment, vascular inflammation, and osteogenic signaling in valvular interstitial cells. Therefore, Lp(a) combines several potentially pathogenic mechanisms, including arterial deposition of an atherogenic lipoprotein particle, OxPL-mediated inflammation, and possible antifibrinolytic effects. This multimodal biology provides a mechanistic basis for the relationship between elevated Lp(a), atherosclerotic cardiovascular disease, and calcific aortic valve disease [4].

### 2.2 Genetic Regulation

Circulating Lp(a) concentrations are primarily determined by genetic variation at the apolipoprotein(a) gene (*LPA*) locus. In particular, the number of kringle IV type 2 repeats strongly influences apo(a) isoform size, with smaller isoforms generally associated with higher circulating Lp(a) concentrations. Although Lp(a) levels remain relatively stable throughout life, substantial interindividual and ancestry-related variability exists. Kidney dysfunction, liver disease, hormonal status, and systemic inflammatory conditions may also modify circulating concentrations, although their influence is considerably smaller than that of inherited genetic determinants. Genetic association studies and Mendelian randomization analyses support a causal role for elevated Lp(a) in atherosclerotic cardiovascular disease (ASCVD) and calcific aortic valve disease [2,5,6].

Lifestyle interventions have little effect on Lp(a) concentrations, and most currently available lipid-lowering therapies do not specifically target its production. Statins generally do not reduce Lp(a) and may modestly increase its concentration, whereas ezetimibe has no consistent clinically meaningful effect. Therapies directed against proprotein convertase subtilisin/kexin type 9 (PCSK9), including monoclonal antibodies and inclisiran, reduce Lp(a) only modestly compared with their pronounced effect on low-density lipoprotein cholesterol. Inclisiran is a small interfering RNA (siRNA) agent that inhibits hepatic PCSK9 synthesis rather than directly targeting the *LPA* gene. Overall, PCSK9-directed therapies generally reduce Lp(a) by approximately 15–30%, an effect that is substantially smaller than that achieved with emerging Lp(a)-specific agents [7,8].

### 2.3 Measurement and Clinical Interpretation of Lp(a)

Accurate measurement of Lp(a) is essential for its incorporation into cardiovascular risk assessment. Lp(a) may be reported as mass concentration in milligrams per deciliter (mg/dL) or as particle concentration in nanomoles per liter (nmol/L). Because the relationship between particle mass and particle number varies according to apo(a) isoform size, these units are not directly interchangeable, and the use of a fixed conversion factor may result in clinically relevant misclassification. Whenever possible, assays that minimize sensitivity to apo(a) isoform size and are traceable to internationally recognized reference material should be preferred [2].

Cardiovascular risk increases progressively across the Lp(a) concentration range, without a single biological threshold separating normal from abnormal values. Nevertheless, concentrations of at least 50 mg/dL or 125 nmol/L are commonly used as pragmatic thresholds to identify individuals at increased cardiovascular risk. These values should be regarded as separate decision thresholds rather than mathematically equivalent measurements. Interpretation should also consider the assay used, ancestry, baseline cardiovascular risk, and the presence of premature or recurrent atherosclerotic cardiovascular disease [2].

## 3. Clinical Implications and Strength of Evidence

Elevated lipoprotein(a) has been associated with a broad spectrum of cardiovascular phenotypes through atherogenic, inflammatory, prothrombotic, and calcific pathways. However, the strength and nature of the supporting evidence are not uniform. Genetic association studies and Mendelian randomization analyses support a causal role for elevated Lp(a) in atherosclerotic cardiovascular disease and calcific aortic valve disease, whereas the evidence for several other clinical conditions is based predominantly on observational associations or indirect mechanistic pathways [2,5,6,9,10].

Accordingly, the following subsections distinguish established causal relationships from associations that remain less certain. This distinction is clinically relevant because an association between elevated Lp(a) and a cardiovascular phenotype does not necessarily demonstrate that pharmacological Lp(a) lowering will improve outcomes in that specific condition.

### 3.1 Coronary Artery Disease

Elevated Lp(a) is causally associated with coronary artery disease and contributes to risk independently of conventional lipid parameters. Its pathogenic effects reflect the combination of cholesterol deposition within the arterial wall, oxidized phospholipid-mediated inflammation, and potential interference with fibrinolytic pathways. The relationship between Lp(a) concentration and coronary risk is continuous rather than dichotomous, with a progressively greater risk observed at higher circulating concentrations [2,5,6].

The clinical relevance of Lp(a) is particularly evident in individuals with premature coronary artery disease, recurrent ischemic events, or progressive atherosclerosis despite intensive low-density lipoprotein cholesterol lowering. In these settings, elevated Lp(a) may identify an important component of residual cardiovascular risk and support more intensive management of all modifiable risk factors. However, until dedicated outcome trials are completed, the extent to which selective Lp(a) reduction will decrease recurrent coronary events remains uncertain [7,8,11].

### 3.2 Ischemic Stroke

Elevated Lp(a) is associated with an increased risk of ischemic stroke, although this relationship appears less consistent than that observed for coronary artery disease and varies according to stroke subtype. The strongest association has been reported for large-artery atherosclerotic stroke, particularly in younger individuals and in patients with premature cerebrovascular atherosclerosis. Observational and genetic studies support a relationship between higher Lp(a) concentrations and ischemic stroke risk [12,13].

Although the structural homology between apolipoprotein(a) and plasminogen has raised the possibility of impaired fibrinolysis, the clinical relevance of this mechanism remains uncertain. Available evidence suggests that the cerebrovascular risk associated with Lp(a) is predominantly mediated by atherosclerotic pathways rather than by a generalized prothrombotic effect. Accordingly, Lp(a) measurement may be particularly informative in patients with premature, recurrent, or otherwise unexplained large-artery ischemic stroke, whereas evidence regarding cardioembolic and small-vessel stroke remains limited [12,13].

### 3.3 Peripheral Artery Disease

Elevated Lp(a) is associated with peripheral artery disease (PAD), reflecting the involvement of peripheral arterial territories in systemic atherosclerosis. Prospective observational studies have linked higher Lp(a) concentrations to prevalent and incident lower-extremity PAD, while genetic analyses support a causal contribution of Lp(a) to atherosclerotic disease in the peripheral circulation. However, the magnitude of this association may vary according to ancestry, population characteristics, and the definition of PAD used in individual studies [14,15].

From a clinical perspective, elevated Lp(a) may be particularly relevant in patients with premature PAD or polyvascular atherosclerotic disease, in whom it may identify an additional component of residual cardiovascular risk. Nevertheless, evidence that Lp(a) independently predicts limb-specific outcomes after PAD has become established remains limited and inconsistent. Moreover, no randomized trial has yet demonstrated that selective Lp(a) lowering reduces major adverse limb events, amputations, or the need for peripheral revascularization [16].

### 3.4 Abdominal Aortic Aneurysm

Elevated Lp(a) has been associated with abdominal aortic aneurysm (AAA) in observational and prospective population studies. More recent analyses combining circulating Lp(a) concentrations with *LPA* genetic variants have strengthened this association and suggest that elevated Lp(a) may contribute to AAA development rather than merely reflect concomitant atherosclerotic disease. However, the biological mechanisms underlying this relationship remain incompletely understood and may involve oxidized phospholipid-mediated inflammation, vascular wall injury, and extracellular matrix remodeling [17,18].

The available evidence relates predominantly to abdominal rather than thoracic aortic aneurysm, and the findings should not be generalized to all forms of aneurysmal or dissecting aortic disease. Furthermore, it remains unknown whether selective Lp(a) lowering can prevent AAA formation, reduce aneurysm expansion, or decrease the risk of rupture and surgical repair. Therefore, elevated Lp(a) should currently be considered an emerging risk factor for AAA rather than an established therapeutic target in this setting [17,19].

### 3.5 Calcific Aortic Valve Disease

Among the cardiovascular phenotypes associated with elevated Lp(a), calcific aortic valve disease (CAVD) is supported by particularly strong genetic and mechanistic evidence. Variants at the LPA locus associated with higher circulating Lp(a) concentrations are also associated with aortic valve calcification and clinically significant aortic stenosis (AS), supporting a causal contribution of Lp(a) to disease initiation. Mechanistically, Lp(a)-associated oxidized phospholipids promote valvular inflammation, osteogenic differentiation of valvular interstitial cells, and progressive mineralization of the valve leaflets [9,10,20].

In patients with established AS, higher concentrations of Lp(a) and oxidized phospholipids carried by apolipoprotein B have been associated with faster hemodynamic progression, greater valvular calcification, and a higher likelihood of aortic valve replacement. However, these observational findings do not establish that pharmacological Lp(a) lowering will necessarily slow disease progression. The ongoing Lp(a)FRONTIERS CAVS trial is evaluating whether pelacarsen can reduce the progression of mild-to-moderate CAVD in patients with elevated Lp(a), but no definitive therapeutic benefit should be assumed before randomized trial results become available [9,21].

### 3.6 Heart Failure

Elevated Lp(a) has been associated with the development of heart failure (HF) in large population-based and genetic studies. However, this relationship appears to be mediated, at least in part, by upstream cardiovascular conditions, particularly coronary artery disease, myocardial infarction, and calcific aortic valve disease, which may ultimately lead to ventricular dysfunction. Evidence supporting a direct myocardial effect of Lp(a), independent of ischemic or valvular disease, remains limited [22,23].

In patients with established HF, observational studies suggest that higher Lp(a) concentrations may be associated with an increased risk of cardiovascular death or HF hospitalization, although the available findings remain limited and may vary according to HF etiology and the burden of concomitant cardiovascular disease. No randomized trial has yet demonstrated that selective Lp(a) lowering prevents the development of HF or improves outcomes in patients with established HF. Therefore, Lp(a) should currently be considered a potential contributor to HF risk, predominantly through associated atherosclerotic and valvular pathways, rather than a validated HF-specific therapeutic target [24].

## 4. Lp(a)-Lowering Therapies in Clinical Development

The development of RNA-targeted therapies, including antisense oligonucleotides (ASOs) and siRNA agents, has transformed the therapeutic landscape of Lp(a), with several compounds achieving reductions exceeding 80% in early-phase clinical trials. Oral small-molecule inhibitors and gene-editing approaches are also being investigated. However, whether profound and sustained Lp(a) lowering translates into a clinically meaningful reduction in cardiovascular events remains to be established by ongoing cardiovascular outcome trials (CVOTs) [25,26,27].

Several mechanistically distinct approaches are currently being evaluated:

• Antisense oligonucleotide therapy: pelacarsen.

• Small interfering RNA therapies: olpasiran, zerlasiran, and lepodisiran.

• Oral small-molecule inhibitor of Lp(a) assembly: muvalaplin.

• *In vivo* gene-editing therapy targeting LPA: CTX320.

These investigational approaches directly reduce apo(a) synthesis, prevent Lp(a) particle assembly, or disrupt LPA at the genomic level. They should be distinguished from therapies developed primarily for other lipid targets that produce secondary reductions in Lp(a). For example, obicetrapib, a cholesteryl ester transfer protein (CETP) inhibitor developed primarily for LDL-C lowering, has also reduced Lp(a) in clinical trials; however, it is not an Lp(a)-specific therapy, and its clinical effects cannot currently be attributed specifically to Lp(a) lowering [28]. The principal Lp(a)-lowering agents, their mechanisms, magnitude of Lp(a) reduction, and current development status are summarized in Table 1 (Ref. [25,26,29,30,31,32,33,34,35,36,37,38,39,40,41,42]).

**Table 1.** **Principal investigational therapies specifically targeting lipoprotein(a)**.

Agent	Modality and route	Molecular mechanism	Key Lp(a)-lowering evidence	Most advanced clinical development	References
Pelacarsen	GalNAc-conjugated ASO; SC	RNase H1-mediated degradation of hepatic *LPA* mRNA, reducing apo(a) synthesis	Dose-dependent mean reductions of approximately 35–80% in phase 2	Phase 3 Lp(a)HORIZON CVOT ongoing	[25,29,30]
Olpasiran	GalNAc-conjugated siRNA; SC	RISC-mediated cleavage of hepatic *LPA* mRNA, reducing apo(a) synthesis	Placebo-adjusted reductions exceeding 95% with doses ≥75 mg every 12 weeks in OCEAN(a)-DOSE	Phase 3 OCEAN(a)-Outcomes and OCEAN(a)-PreEvent ongoing	[26,31,32,33]
Zerlasiran	GalNAc-conjugated siRNA; SC	RISC-mediated cleavage of hepatic *LPA* mRNA, reducing apo(a) synthesis	Placebo-adjusted time-averaged reductions of 81.3–85.6% through week 36 in ALPACAR-360	Phase 2 completed; phase 3-ready, but no phase 3 CVOT initiated	[34]
Lepodisiran	GalNAc-conjugated extended-duration siRNA; SC	RISC-mediated cleavage of hepatic *LPA* mRNA, reducing apo(a) synthesis	93.9% placebo-adjusted time-averaged reduction from days 60–180 with the pooled 400 mg regimens in ALPACA	Phase 3 ACCLAIM-Lp(a) ongoing	[35,36]
Muvalaplin	Oral small molecule; once daily	Binds apo(a) and prevents the initial apo(a)–apoB-100 interaction and intact Lp(a) particle assembly	At week 12, reductions of up to 85.8% with the intact-particle assay and 70.0% with the apo(a)-based assay in KRAKEN	Phase 3 MOVE-Lp(a) ongoing	[37,38,39]
CTX320	*In vivo* CRISPR-Cas9 gene-editing; single IV infusion	LNP delivery of Cas9 mRNA and an LPA-specific guide RNA to disrupt hepatic *LPA*	Reduction of up to 73% during phase 1 dose escalation; preliminary sponsor-reported, non-peer-reviewed data	First-in-human phase 1 trial ongoing	[40,41,42]

Reported reductions are placebo-adjusted values from key phase 1 or phase 2 studies unless otherwise specified. Direct comparisons among agents should be avoided because the trials differed in study populations, baseline Lp(a) concentrations, assay methods, dosing schedules, follow-up duration, and statistical analyses. The CTX320 efficacy estimate is preliminary, sponsor-reported, and has not yet been published in a peer-reviewed report. Development status was updated in July 2026. apo(a), apolipoprotein(a); apoB-100, apolipoprotein B-100; ASO, antisense oligonucleotide; CRISPR-Cas9, clustered regularly interspaced short palindromic repeats-CRISPR-associated protein 9; CVOT, cardiovascular outcome trial; GalNAc, N-acetylgalactosamine; IV, intravenous; LPA, gene encoding apolipoprotein(a); LNP, lipid nanoparticle; Lp(a), lipoprotein(a); mRNA, messenger RNA; RISC, RNA-induced silencing complex; SC, subcutaneous; siRNA, small interfering RNA. Lp(a) reductions are reported as percentage change from baseline unless otherwise specified. Values indicated as placebo-adjusted represent the placebo-adjusted percentage change from baseline, as reported in the corresponding trials.

## 5. Pelacarsen: Antisense Oligonucleotide Targeting Apolipoprotein(a)

### 5.1 Mechanism of Action

Pelacarsen is a second-generation, N-acetylgalactosamine-conjugated antisense oligonucleotide designed to selectively bind LPA messenger RNA in hepatocytes. Formation of the antisense oligonucleotide–messenger RNA complex promotes RNase H1-mediated degradation of LPA messenger RNA, thereby reducing hepatic apolipoprotein(a) synthesis and limiting the assembly of circulating Lp(a) particles. Conjugation with N-acetylgalactosamine facilitates receptor-mediated uptake by hepatocytes and increases target specificity and potency compared with earlier unconjugated antisense constructs [25].

### 5.2 Lp(a)-Lowering Efficacy and Safety

In a randomized, double-blind, dose-ranging phase 2 trial involving 286 patients with established cardiovascular disease and elevated Lp(a), pelacarsen produced dose-dependent mean reductions in circulating Lp(a) ranging from approximately 35% to 80%. The greatest reduction was observed with the 20 mg weekly regimen. Injection-site reactions were the most frequent adverse events and occurred more commonly with pelacarsen than with placebo, although they were generally mild. This study established proof of biological efficacy but was not designed or powered to determine whether pelacarsen reduces cardiovascular events [25].

More recently, the randomized Lp(a)FRONTIERS APHERESIS trial evaluated pelacarsen 80 mg administered subcutaneously every four weeks in patients with established cardiovascular disease who were receiving regular lipoprotein apheresis because of markedly elevated Lp(a). At week 52, pelacarsen produced a placebo-adjusted Lp(a) reduction of 72% when measured in mg/dL and substantially reduced the number of required apheresis sessions. Injection-site reactions were more frequent with pelacarsen and were predominantly mild. Although these findings support the efficacy of monthly pelacarsen in a highly selected population, the trial was small and did not assess cardiovascular outcomes [29].

### 5.3 Lp(a)HORIZON Cardiovascular Outcome Trial

Lp(a)HORIZON (NCT04023552) is a randomized, double-blind, placebo-controlled phase 3 cardiovascular outcome trial evaluating pelacarsen 80 mg administered subcutaneously once monthly in more than 8300 patients with established cardiovascular disease and baseline Lp(a) concentrations of at least 70 mg/dL. Eligible cardiovascular disease includes previous myocardial infarction, ischemic stroke, or clinically significant symptomatic peripheral artery disease. The primary endpoint is a composite of cardiovascular death, nonfatal myocardial infarction, nonfatal stroke, or urgent coronary revascularization requiring hospitalization [30].

The primary endpoint will be evaluated both in the overall trial population and in the prespecified subgroup with baseline Lp(a) concentrations of at least 90 mg/dL. At the time of this revision, no trial results had been posted. Lp(a)HORIZON is therefore expected to provide the first definitive randomized evidence regarding whether selective pharmacological lowering of Lp(a) reduces major cardiovascular events [30].

### 5.4 Additional Clinical Development and Potential Positioning

A separate randomized phase 3 study (NCT06813911) is evaluating the efficacy, safety, and tolerability of pelacarsen in patients with established atherosclerotic cardiovascular disease, Lp(a) concentrations of at least 175 nmol/L, and low-density lipoprotein cholesterol above 70 mg/dL despite background inclisiran therapy. This study is designed to assess the feasibility of simultaneously targeting residual risk related to elevated Lp(a) and low-density lipoprotein cholesterol; it should not, however, be interpreted as a cardiovascular outcome trial [43].

If Lp(a)HORIZON demonstrates a favorable balance of cardiovascular efficacy and safety, pelacarsen could become an Lp(a)-specific treatment for patients with established atherosclerotic cardiovascular disease and markedly elevated Lp(a). Its eventual clinical positioning will depend on the magnitude of absolute and relative event reduction, long-term safety, regulatory indications, treatment burden, cost-effectiveness, and the identification of patients with the greatest expected absolute benefit.

## 6. Olpasiran: Small Interfering RNA Targeting Apolipoprotein(a)

### 6.1 Mechanism of Action

Olpasiran is a hepatocyte-directed siRNA designed to selectively suppress hepatic apolipoprotein(a) synthesis. Following cellular uptake, the antisense strand of olpasiran is incorporated into the RNA-induced silencing complex (RISC), which recognizes and cleaves complementary LPA messenger RNA. This process reduces the availability of messenger RNA for apolipoprotein(a) translation and consequently limits the hepatic assembly of circulating Lp(a) particles. The intracellular persistence of the silencing complex enables prolonged pharmacodynamic activity and supports dosing intervals of several months [26].

### 6.2 Lp(a)-Lowering Efficacy and Safety

OCEAN(a)-DOSE was a randomized, double-blind, placebo-controlled, dose-ranging phase 2 trial involving 281 patients with established atherosclerotic cardiovascular disease and baseline Lp(a) concentrations greater than 150 nmol/L. Participants received subcutaneous olpasiran at doses of 10 mg, 75 mg, or 225 mg every 12 weeks, 225 mg every 24 weeks, or placebo. At week 36, doses of at least 75 mg every 12 weeks produced placebo-adjusted mean reductions in Lp(a) exceeding 95%. More than 98% of participants receiving these regimens achieved Lp(a) concentrations of 125 nmol/L or lower [26].

Overall adverse-event rates were similar between the olpasiran and placebo groups. Injection-site reactions, predominantly mild pain, were the most frequent treatment-related adverse events. However, the trial was designed to evaluate biomarker efficacy, dose selection, and short-term safety rather than cardiovascular outcomes; therefore, the profound reduction in Lp(a) should not be interpreted as evidence of clinical benefit [26].

### 6.3 Durability of Lp(a) Reduction

The off-treatment extension of OCEAN(a)-DOSE demonstrated that the pharmacodynamic effect of olpasiran persisted for several months after the final administration. Participants who had received doses of at least 75 mg every 12 weeks retained an approximately 40–50% placebo-adjusted reduction in Lp(a) nearly one year after the last dose. No new safety signal emerged during the extended observation period. These findings demonstrate prolonged biological activity but also indicate that the effect gradually diminishes after treatment discontinuation [31].

The long duration of action may allow infrequent administration and potentially improve treatment adherence. Nevertheless, the optimal maintenance dose and interval will ultimately depend on the magnitude and consistency of Lp(a) suppression, long-term safety, clinical efficacy, and the dosing regimen evaluated in phase 3 trials.

### 6.4 OCEAN(a)-Outcomes

OCEAN(a)-Outcomes (NCT05581303) is an ongoing randomized, double-blind, placebo-controlled phase 3 cardiovascular outcome trial involving 7297 patients with established atherosclerotic cardiovascular disease and Lp(a) concentrations of at least 200 nmol/L. Eligible participants had a history of myocardial infarction or previous percutaneous coronary intervention accompanied by at least one additional cardiovascular risk factor. The primary endpoint is a composite of coronary heart disease death, myocardial infarction, or urgent coronary revascularization [32].

Enrollment has been completed, and the trial remains ongoing, with estimated completion in March 2028. No outcome results were publicly available at the time of this revision. Consequently, OCEAN(a)-Outcomes will be essential in determining whether the marked and sustained reduction in Lp(a) achieved with olpasiran translates into a reduction in major coronary events [32].

### 6.5 Expansion to Patients Without Previous Major Cardiovascular Events

OCEAN(a)-PreEvent (NCT07136012) is an ongoing randomized, double-blind, placebo-controlled phase 3 trial evaluating olpasiran in approximately 11,000 individuals aged 50 years or older with Lp(a) concentrations of at least 200 nmol/L who are at high risk for a first major cardiovascular event. Eligibility is based on the presence of multiple cardiovascular risk factors and/or evidence of atherosclerosis, while individuals with previous myocardial infarction, stroke, transient ischemic attack, acute limb ischemia, or arterial revascularization are excluded. The primary endpoint is a composite of coronary heart disease death, myocardial infarction, or urgent coronary revascularization [33].

This trial extends the olpasiran development program beyond conventional secondary prevention and may clarify whether selective Lp(a) lowering is beneficial before the occurrence of a first major cardiovascular event. However, its results are not expected for several years, and no clinical benefit in this population can currently be assumed [33].

## 7. Additional Small Interfering RNA Therapies

### 7.1 Zerlasiran

Zerlasiran is a hepatocyte-directed small interfering RNA designed to suppress hepatic LPA messenger RNA and reduce apolipoprotein(a) synthesis. In the randomized, double-blind, placebo-controlled phase 2 ALPACAR-360 trial, 178 patients with stable atherosclerotic cardiovascular disease and baseline Lp(a) concentrations of at least 125 nmol/L received zerlasiran 450 mg every 24 weeks, 300 mg every 16 weeks, 300 mg every 24 weeks, or placebo. The placebo-adjusted time-averaged reductions in Lp(a) from baseline through week 36 were 85.6%, 82.8%, and 81.3%, respectively [34].

Lp(a) lowering remained substantial during extended follow-up, although concentrations gradually increased after the final administration. The most frequently reported treatment-related adverse events were injection-site reactions, which were generally mild. ALPACAR-360 was designed to assess Lp(a) reduction and safety rather than cardiovascular events; therefore, these findings do not establish that zerlasiran improves clinical outcomes [34].

### 7.2 Lepodisiran

Lepodisiran is an extended-duration small interfering RNA targeting hepatic LPA messenger RNA. The randomized, double-blind, placebo-controlled phase 2 ALPACA trial included 320 participants with elevated Lp(a). Participants received lepodisiran 16 mg, 96 mg, or 400 mg at baseline and again at day 180; a separate group received 400 mg at baseline followed by placebo at day 180. From day 60 to day 180, the placebo-adjusted time-averaged reductions in Lp(a) were 40.8% with 16 mg, 75.2% with 96 mg, and 93.9% in the pooled 400 mg groups [35].

The pharmacodynamic effect was prolonged. From day 30 to day 360, the placebo-adjusted time-averaged reduction was 88.5% among participants who received a single 400 mg dose and 94.8% among those who received a second 400 mg dose at day 180. Injection-site reactions occurred more frequently with lepodisiran than with placebo, although no clear excess of serious adverse events was observed. As with the other phase 2 studies, ALPACA was not designed to establish cardiovascular efficacy [35].

### 7.3 ACCLAIM-Lp(a) Cardiovascular Outcome Trial

ACCLAIM-Lp(a) (NCT06292013) is a randomized, double-blind, placebo-controlled phase 3 cardiovascular outcome trial evaluating lepodisiran in adults with Lp(a) concentrations of at least 175 nmol/L. The study includes both patients with established atherosclerotic cardiovascular disease and individuals at high risk for a first cardiovascular event. The primary efficacy assessment is based on the time to first occurrence of a four-component major adverse cardiovascular event composite, including cardiovascular death, nonfatal myocardial infarction, nonfatal ischemic stroke, or urgent coronary revascularization [36].

Following an expansion of the study population and dosing program, the planned enrollment is approximately 17,300 participants. Enrollment has been completed, and the study remains active, with estimated completion in 2029. ACCLAIM-Lp(a) will therefore provide outcome evidence across a broader risk spectrum than trials restricted exclusively to conventional secondary prevention populations [36].

## 8. Muvalaplin: Oral Small-Molecule Inhibitor of Lp(a) Assembly

### 8.1 Mechanism of Action

Muvalaplin is an orally administered small molecule designed to inhibit the assembly of Lp(a) particles. Lp(a) formation begins with a noncovalent interaction between kringle IV domains 7 and 8 of apolipoprotein(a) and lysine residues on apolipoprotein B-100, followed by formation of a covalent disulfide bond. Muvalaplin binds to apolipoprotein(a) and disrupts this initial interaction, thereby preventing the subsequent formation of intact Lp(a) particles [37].

This mechanism differs fundamentally from that of antisense oligonucleotide and small interfering RNA therapies, which reduce hepatic apolipoprotein(a) synthesis. Because apolipoprotein(a) shares structural homology with plasminogen, interference with plasminogen activity represents a potential theoretical concern. However, no clinically relevant reduction in plasminogen activity was observed in the phase 1 or phase 2 studies conducted to date [37,38].

### 8.2 Phase 1 Evidence

In a randomized phase 1 trial involving healthy participants with or without elevated Lp(a), once-daily muvalaplin administered for 14 days produced dose-dependent reductions in circulating Lp(a), reaching approximately 65% at the highest tested dose. Treatment was generally well tolerated, and no clinically relevant effect on plasminogen activity was observed. These preliminary findings supported longer-duration evaluation in patients at increased cardiovascular risk [37].

### 8.3 KRAKEN Phase 2 Trial

KRAKEN (NCT05563246) was a randomized, double-blind, placebo-controlled phase 2 trial involving 233 adults with Lp(a) concentrations of at least 175 nmol/L and established atherosclerotic cardiovascular disease, diabetes mellitus, or familial hypercholesterolemia. Participants received oral muvalaplin at doses of 10 mg, 60 mg, or 240 mg once daily, or placebo, for 12 weeks [38].

At week 12, placebo-adjusted reductions in Lp(a) measured using an assay specific for intact Lp(a) particles were 47.6%, 81.7%, and 85.8% with the 10 mg, 60 mg, and 240 mg doses, respectively. Using a conventional apolipoprotein(a)-based assay, the corresponding reductions were 40.4%, 70.0%, and 68.9%. The different estimates reflect the mechanism of muvalaplin: conventional assays may also detect free apolipoprotein(a) or apolipoprotein(a) bound to the drug, whereas the intact-particle assay identifies apolipoprotein(a) associated with an apolipoprotein B-containing particle [38].

Adverse-event rates were similar across the treatment and placebo groups, with no dose-dependent safety or tolerability signal and no meaningful effect on plasminogen activity. Muvalaplin also produced modest dose-dependent reductions in apolipoprotein B and low-density lipoprotein cholesterol. Nevertheless, KRAKEN was limited to 12 weeks of treatment and was not designed to evaluate cardiovascular outcomes or long-term safety [38].

### 8.4 MOVE-Lp(a) Cardiovascular Outcome Trial

MOVE-Lp(a) (NCT07157774) is an ongoing randomized, double-blind, placebo-controlled phase 3 cardiovascular outcome trial evaluating whether oral muvalaplin reduces major adverse cardiovascular events in adults with Lp(a) concentrations of at least 175 nmol/L. The study includes patients who have experienced a previous atherosclerotic cardiovascular event as well as selected individuals at high risk for a first event [39].

The trial is expected to enroll approximately 10,450 participants and is currently recruiting, with estimated completion in 2031. MOVE-Lp(a) represents the first large cardiovascular outcome trial of an orally administered inhibitor of Lp(a) assembly. Until its results are available, the substantial biomarker reductions observed in KRAKEN should not be interpreted as evidence that muvalaplin prevents cardiovascular events [39].

### 8.5 Potential Clinical Positioning

Oral administration distinguishes muvalaplin from the injectable antisense oligonucleotide and small interfering RNA therapies currently in development. A once-daily oral treatment may offer practical advantages for some patients, although these must be weighed against the prolonged dosing intervals achievable with RNA-based therapies. The eventual clinical role of muvalaplin will depend on cardiovascular efficacy, long-term safety, treatment adherence, drug interactions, cost, and the reliability and availability of assays capable of accurately measuring intact Lp(a) during treatment.

## 9. CTX320: *In Vivo* CRISPR-Cas9 Gene-Editing Targeting LPA

### 9.1 Mechanism and Therapeutic Rationale

CTX320 is an investigational *in vivo* gene-editing therapy designed to disrupt the hepatic LPA gene and thereby reduce apolipoprotein(a) production. The treatment consists of Cas9 messenger RNA and an LPA-specific guide RNA encapsulated within lipid nanoparticles. Following intravenous administration, the lipid nanoparticles deliver the gene-editing components predominantly to hepatocytes, where the guide RNA directs Cas9 to a selected genomic sequence within LPA. Repair of the resulting DNA double-strand break through error-prone non-homologous end joining is intended to introduce insertions or deletions that impair functional apolipoprotein(a) synthesis [40].

Unlike antisense oligonucleotide and small interfering RNA therapies, which require repeated administration to maintain suppression of apolipoprotein(a), CTX320 is intended to produce prolonged or potentially permanent Lp(a) lowering after a single treatment. However, the durability and completeness of LPA disruption in humans have not yet been established, and the irreversible nature of genomic editing creates safety and regulatory considerations that differ fundamentally from those of reversible pharmacological therapies [40].

### 9.2 First-in-Human Phase 1 Trial

CTX320 is being evaluated in a multicenter, open-label, ascending single-dose phase 1 trial (ACTRN12623001095651) involving patients with elevated Lp(a) and either stable atherosclerotic cardiovascular disease or mild-to-moderate calcific aortic valve disease. Participants receive a single intravenous infusion of CTX320, with planned dose levels ranging from 0.1 to 0.8 mg/kg. The principal objectives are to assess dose-limiting toxicities, overall safety and tolerability, pharmacokinetics, and changes in circulating Lp(a), and to identify a dose suitable for further clinical development [41].

The study is uncontrolled and was initially designed to enroll approximately 24 participants. Consequently, it cannot determine whether CTX320 reduces cardiovascular events or modifies the progression of calcific aortic valve disease. The trial also includes clinical and laboratory follow-up for at least 12 months after treatment, although substantially longer surveillance will be required to characterize the durability and long-term safety of an irreversible gene-editing intervention [41].

### 9.3 Preliminary Clinical Evidence

Preliminary sponsor-reported findings from the dose-escalation phase indicated reductions in circulating Lp(a) of up to 73% following a single administration of CTX320. However, detailed information regarding the number and clinical characteristics of treated participants, dose-specific responses, duration of follow-up, variability of Lp(a) reduction, and complete safety findings has not yet been published in a peer-reviewed report [42].

These preliminary observations therefore provide only an initial signal of biological activity and should not be directly compared with the efficacy estimates derived from randomized phase 2 trials of antisense oligonucleotide or small interfering RNA therapies. Confirmation in larger studies with longer follow-up, standardized Lp(a) assays, and independent peer-reviewed reporting will be required before the magnitude and durability of the treatment effect can be reliably established [42].

### 9.4 Safety Considerations and Future Development

The potential advantages of a one-time intervention must be balanced against uncertainties inherent to *in vivo* genome editing. Relevant issues include unintended editing at off-target genomic sites, large or unexpected on-target genomic alterations, immune responses to the Cas9 protein or lipid nanoparticle components, transient or persistent hepatic injury, interindividual variability in editing efficiency, and the inability to readily reverse the intervention once administered. Long-term surveillance will therefore be essential even if early biochemical efficacy and short-term tolerability appear favorable.

CTX320 remains an early-stage investigational therapy and should not yet be considered a clinical alternative to pharmacological Lp(a)-lowering agents. Its future development will depend on the reproducibility and durability of Lp(a) reduction, the absence of clinically important off-target or hepatic safety signals, and evidence that permanent gene disruption offers a favorable benefit–risk balance in appropriately selected patients with substantial lifetime cardiovascular risk. The designs and current status of the principal ongoing randomized trials evaluating Lp(a)-lowering therapies are summarized in Table 2 (Ref. [21,30,32,33,36,39,43]).

**Table 2.** **Principal ongoing randomized trials of Lipoprotein(a)-lowering therapies**.

Trial and registration	Agent and phase	Population	Primary objective or endpoint	Enrollment and current status	References
Lp(a)HORIZON (NCT04023552)	Pelacarsen; phase 3 CVOT	Established cardiovascular disease and Lp(a) ≥70 mg/dL	Time to first cardiovascular death, nonfatal myocardial infarction, nonfatal stroke, or urgent coronary revascularization requiring hospitalization	8323 participants; active, not recruiting; no results posted at the time of revision	[30]
OCEAN(a)-Outcomes (NCT05581303)	Olpasiran; phase 3 CVOT	Established ASCVD, principally previous myocardial infarction or percutaneous coronary intervention, and Lp(a) ≥200 nmol/L	Time to first coronary heart disease death, myocardial infarction, or urgent coronary revascularization	7297 participants; active, not recruiting; estimated completion March 2028	[32]
ACCLAIM-Lp(a) (NCT06292013)	Lepodisiran; phase 3 CVOT	Established ASCVD or high risk for a first cardiovascular event and Lp(a) ≥175 nmol/L	Time to first occurrence of cardiovascular death, nonfatal myocardial infarction, nonfatal ischemic stroke, or urgent coronary revascularization	Approximately 17,300 participants; active, not recruiting; estimated completion March 2029	[36]
OCEAN(a)-PreEvent (NCT07136012)	Olpasiran; phase 3 CVOT	Adults aged ≥50 years at high risk for a first major cardiovascular event, with Lp(a) ≥200 nmol/L and no previous major atherothrombotic event or arterial revascularization	Time to first coronary heart disease death, myocardial infarction, or urgent coronary revascularization	Approximately 11,000 participants; recruiting; estimated completion October 2031	[33]
MOVE-Lp(a) (NCT07157774)	Muvalaplin; phase 3 CVOT	Previous ASCVD event or high risk for a first ASCVD event and Lp(a) ≥175 nmol/L	Time to first occurrence of cardiovascular death, nonfatal myocardial infarction, nonfatal ischemic stroke, or urgent coronary revascularization	Approximately 10,450 participants; recruiting; estimated completion March 2031	[39]
Lp(a)FRONTIERS CAVS (NCT05646381)	Pelacarsen; phase 2 disease-modification trial	Mild-to-moderate calcific aortic valve stenosis and Lp(a) ≥175 nmol/L	Assessment of calcific aortic valve stenosis progression using echocardiographic and computed-tomography measures, including peak aortic jet velocity and aortic valve calcium score	Approximately 502 participants; recruiting; estimated completion March 2030	[21]
ADD-VANTAGE (NCT06813911)	Pelacarsen on background inclisiran; phase 3 efficacy and safety trial	Established ASCVD, elevated Lp(a), and LDL-C above the protocol threshold despite background inclisiran	Evaluation of Lp(a)-lowering efficacy, safety, and tolerability; not a cardiovascular outcome trial	Approximately 340 participants; recruiting; estimated completion February 2028	[43]

Trial status and estimated completion dates were updated in July 2026 and may change during study conduct. Lp(a)HORIZON, OCEAN(a)-Outcomes, ACCLAIM-Lp(a), OCEAN(a)-PreEvent, and MOVE-Lp(a) are cardiovascular outcome trials. Lp(a)FRONTIERS CAVS evaluates the progression of calcific aortic valve stenosis, whereas ADD-VANTAGE primarily evaluates the efficacy, safety, and tolerability of pelacarsen on background inclisiran. ASCVD, atherosclerotic cardiovascular disease; CVOT, cardiovascular outcome trial; LDL-C, low-density lipoprotein cholesterol; Lp(a), lipoprotein(a).

## 10. Translational Implications and Potential Biomarkers

### 10.1 Translational Significance

The immediate translational relevance of Lp(a) lies in its ability to refine cardiovascular risk assessment and identify individuals whose inherited risk may not be adequately represented by conventional lipid measurements. Measurement of Lp(a) at least once in adulthood may be particularly informative in patients with premature or recurrent atherosclerotic cardiovascular disease, a family history of premature cardiovascular events, familial hypercholesterolemia, calcific aortic valve disease, or an unexpectedly high cardiovascular burden despite apparently well-controlled conventional risk factors [2,44].

Identification of elevated Lp(a) should currently prompt comprehensive optimization of all modifiable cardiovascular risk factors, including low-density lipoprotein cholesterol, blood pressure, glycemic control, smoking, body weight, and physical activity. Although these interventions have little direct effect on Lp(a) concentrations, they may reduce the overall absolute risk associated with lifelong exposure to elevated Lp(a). Cascade testing of first-degree relatives may also identify family members with previously unrecognized inherited risk [2,44].

The availability of highly effective Lp(a)-lowering agents may eventually transform Lp(a) measurement from a risk-stratification tool into a treatment-selection biomarker. However, this transition requires evidence that lowering Lp(a) reduces cardiovascular events, determination of the patients who derive the greatest absolute benefit, and definition of clinically relevant treatment thresholds and targets.

### 10.2 Potential Biomarkers and Multimodal Phenotyping

Circulating Lp(a) concentration remains the principal clinically available biomarker for identifying Lp(a)-related cardiovascular risk and for assessing the pharmacodynamic response to targeted therapies. Nevertheless, the concentration required to achieve a clinically meaningful treatment benefit has not yet been established. Moreover, percentage reductions may not fully represent biological benefit, because the same relative reduction produces substantially different absolute changes in patients with moderately versus extremely elevated baseline concentrations.

Additional biomarkers may provide mechanistic or prognostic information beyond the circulating Lp(a) concentration. Apo(a) isoform size and selected LPA genetic variants are closely related to lifelong exposure but are not routinely required for clinical risk assessment. Oxidized phospholipids measured on apolipoprotein B-containing particles or apo(a) may reflect the proinflammatory cargo of Lp(a), although assay availability, analytical standardization, and evidence supporting treatment-guided use remain limited [4,45].

Imaging may provide complementary phenotypic information rather than a specific measure of Lp(a) activity. Coronary artery calcium scoring may refine atherosclerotic cardiovascular risk assessment in individuals with elevated Lp(a), whereas computed tomography quantification of aortic valve calcium can identify the valvular phenotype associated with elevated Lp(a). However, neither imaging marker is specific to Lp(a), because both are influenced by age and other cardiovascular risk factors. Future studies should determine whether combining circulating Lp(a), oxidized phospholipid-related biomarkers, and imaging measures improves patient selection or predicts response to targeted treatment [46,47].

## 11. Limitations of Current Evidence and Unresolved Questions

Despite strong genetic and epidemiological evidence linking elevated Lp(a) to cardiovascular disease, several limitations affect the interpretation of the existing literature. Observational studies differ substantially in population characteristics, ancestry, baseline cardiovascular risk, outcome definitions, assay methodology, and the units used to report Lp(a). Residual confounding and differences in apo(a) isoform sensitivity may also contribute to variation among reported associations.

Evidence regarding emerging therapies is currently based predominantly on relatively short phase 1 and phase 2 trials designed to assess biomarker efficacy and initial safety. These studies consistently demonstrate marked reductions in circulating Lp(a), but they were not powered to evaluate cardiovascular events. Direct comparisons among agents should therefore be avoided because trials differ in baseline Lp(a) concentrations, dosing schedules, assay methods, follow-up duration, and statistical approaches used to report treatment effects.

Long-term safety also remains incompletely characterized. Relevant uncertainties include repeated exposure to injectable RNA-based therapies, hepatic and immunologic safety, adherence to daily oral treatment, and the durability and reversibility of gene-editing approaches. The available studies have generally included selected trial populations and may not fully represent older patients, individuals with advanced kidney or liver disease, or populations from diverse ancestral backgrounds.

Further questions concern the absolute or relative reduction in Lp(a) required to produce clinical benefit, the baseline concentration at which treatment should be initiated, and whether the benefit will extend from secondary prevention to individuals without previous cardiovascular events. It also remains uncertain whether Lp(a) lowering can modify the progression of calcific aortic valve disease, abdominal aortic aneurysm, or heart failure. These questions can only be addressed through adequately powered randomized outcome trials and dedicated disease-specific studies.

## 12. Future Clinical Positioning

### 12.1 Patients Most Likely to Be Treated Initially

If ongoing cardiovascular outcome trials demonstrate clinical benefit, the first candidates for Lp(a)-lowering therapy will likely be patients with established atherosclerotic cardiovascular disease and markedly elevated Lp(a), particularly those with premature disease, polyvascular involvement, or recurrent events despite intensive management of low-density lipoprotein cholesterol and other modifiable risk factors. In these individuals, the high baseline event rate may translate into a greater absolute treatment benefit.

Treatment in primary prevention will require a more individualized approach. Potential candidates may include individuals with extremely elevated Lp(a), a strong family history of premature cardiovascular disease, familial hypercholesterolemia, or evidence of substantial subclinical atherosclerosis. However, the balance among lifetime risk, absolute short-term benefit, treatment burden, cost, and long-term safety must be established before widespread use in lower-risk populations.

Patients with calcific aortic valve disease represent a biologically compelling but distinct population. Although genetic and mechanistic evidence supports a causal contribution of Lp(a) to disease initiation, treatment should not be recommended specifically to slow valvular progression unless dedicated randomized trials demonstrate efficacy.

### 12.2 Selection Among Therapeutic Platforms

The future selection of an Lp(a)-lowering therapy will depend not only on the magnitude of biomarker reduction but also on cardiovascular outcome efficacy, safety, dosing frequency, reversibility, cost, patient preference, and healthcare-system accessibility. Antisense oligonucleotide and small interfering RNA agents offer targeted hepatic suppression with relatively infrequent administration, whereas oral inhibition of particle assembly may provide greater convenience for patients who prefer non-injectable therapy.

Gene-editing strategies could potentially provide highly durable Lp(a) reduction after a single administration, but their irreversible nature and limited clinical experience will require a particularly favorable long-term benefit–risk profile. Ultimately, these therapeutic platforms may occupy complementary rather than competing roles, with treatment selection adapted to baseline risk, Lp(a) concentration, age, comorbidities, and individual preferences.

Cardiovascular outcome trials will therefore determine not only whether Lp(a) is a modifiable causal risk factor, but also whether the magnitude of clinical benefit justifies systematic screening and long-term targeted treatment. Positive findings would establish a new model of genetically informed cardiovascular prevention, whereas neutral results would require reconsideration of the relationship between biomarker reduction and clinical efficacy.

## 13. Conclusions

Lipoprotein(a) should no longer be regarded as a neglected or merely associative cardiovascular biomarker. Genetic, epidemiological, and mechanistic evidence identifies elevated Lp(a) as a causal contributor to atherosclerotic cardiovascular disease and calcific aortic valve disease. Its measurement can already improve risk stratification, identify inherited cardiovascular risk that may be missed by conventional lipid assessment, and support more intensive control of all modifiable risk factors.

The development of antisense oligonucleotides, small interfering RNA agents, oral inhibitors of Lp(a) assembly, and *in vivo* gene-editing strategies has made profound Lp(a) reduction biologically achievable. The decisive remaining question is whether this reduction prevents cardiovascular events and, potentially, modifies calcific valvular disease. Positive results from ongoing outcome trials would establish Lp(a) as both a causal and therapeutically modifiable cardiovascular risk factor, opening a new era of genetically informed and mechanism-based prevention. Until those results are available, biomarker efficacy must not be equated with clinical benefit, but the direction of the field is clear: Lp(a) is moving from risk recognition toward targeted intervention.

## Declaration of AI and AI-Assisted Technologies in the Writing Process

During the preparation and revision of this work, the authors used ChatGPT (OpenAI) to assist with language editing, grammatical correction, manuscript restructuring, and refinement of selected passages. The tool was not used to generate or analyze original data. After using this tool, the authors critically reviewed, verified, and edited all AI-assisted content, including the scientific statements and references, and take full responsibility for the accuracy, integrity, and content of the publication.
